# Spatial regularities in a closed-loop audiovisual search task bias subsequent free-viewing behavior

**DOI:** 10.3758/s13423-025-02703-8

**Published:** 2025-07-07

**Authors:** Sebastiano Cinetto, Elvio Blini, Andrea Zangrossi, Maurizio Corbetta, Marco Zorzi

**Affiliations:** 1 Department of General Psychology and Padova Neuroscience Center, University of Padua, Padua, Italy; 2 Department of Neuroscience, Psychology, Pharmacology and Child Health, University of Florence, Florence, Italy; 3 Department of Neuroscience and Padova Neuroscience Center, University of Padua, Padua, Italy; 4 IRCCS San Camillo Hospital, Venice, Italy

**Keywords:** Eye movements and visual attention, Statistical learning, Free viewing, Spatial attention

## Abstract

Statistical learning of spatial regularities during visual search leads to prioritization of target-rich locations. The resulting attentional bias may subsequently affect orienting and search behavior in similar tasks but its transfer to free viewing has not been demonstrated. We exploited a novel closed-loop paradigm where human observers searched for invisible target locations on a screen only guided by real-time auditory feedback conveying gaze-target distance. Unbeknownst to participants, location probability was biased towards one hemifield. Free viewing during rest, free image viewing, and spatial judgments were assessed before and after the search task. Search performance systematically improved and peaked in the biased hemifield, showing the unfolding of statistical learning. Importantly, the spatial bias transferred to both free-viewing conditions in terms of mean horizontal fixation position, while it did not transfer to spatial judgments. Exploratory results suggest that search performance was influenced by participants’ viewing pattern, whereas transfer was modulated by pre-existing (natural) spatial biases. Our results demonstrate that task-based statistical learning transfers to ecological scenarios, paving the way for future research and clinical applications aimed at ameliorating pathological spatial biases.

**Supplementary Information:**

The online version contains supplementary material available at 10.3758/s13423-025-02703-8.

## Introduction

Our brain possesses the remarkable ability to capture patterns unintentionally within our environment. This phenomenon was termed *statistical learning* in a seminal study by Saffran et al. (1996), where they observed that infants as young as 8 months old could detect recurring syllables within a stream of artificial speech. Subsequent investigations have documented statistical learning across various modalities and domains, including non-linguistic sounds (Saffran et al., 1999), visual scenes (Fiser & Aslin, 2001), tactile stimuli (Conway & Christiansen, 2005), and spatial locations (Mayr, 1996). Although statistical learning was initially regarded as a unitary phenomenon, later studies discovered the recruitment of different brain networks across domains and modalities, and the related intraindividual behavioral variability (Frost et al., 2019). Accordingly, over the past decade, statistical learning has been recognized as a multifaceted process involving domain-specific regions (e.g., visual, auditory, somatosensory, motor cortex) as well as brain areas linked to domain-general processing (e.g., medial temporal lobe, striatum, prefrontal cortex) (Conway, 2020; Frost et al., 2019). Critically, one aspect that remains poorly understood is the potential for transfer of statistical learning across tasks. The limited transfer could be due to the preferential recruitment of domain specific over domain general mechanisms during the learning phase which eventually favors transfer only to similar tasks (Conway, 2020).

A type of statistical learning that pertains to visual exploration is “location probability learning” (Geng & Behrmann, 2002; Jiang, 2018; Jiang, Swallow, & Capistrano et al., 2013a; Miller, 1988). This refers to the finding that spatial locations that frequently contain a visual search target are prioritized in attentional allocation and search behavior. This phenomenon is relatively long-lasting (up to a week; Jiang, Swallow, & Capistrano et al., 2013a, Jiang, Swallow, & Rosenbaum et al., 2013b) and resilient to aging (Twedell et al., 2017) as well as to neurological impairments (Geng & Behrmann, 2002; Sisk et al., 2018). Moreover, most participants show bias without becoming fully aware of the biased spatial distribution of target stimuli (Jiang et al., 2018). While these findings suggest that the spatial bias induced by the location probability manipulation can influence visual processing and attention orienting, its transfer to other visuospatial tasks or to free viewing is largely unexplored. A few studies suggest transfer to related tasks, such as searching for letters among distractors (Ts among Ls) or specific objects in a visual scene (Jiang et al., 2015; Salovich et al., 2018). However, transfer to unrelated, ecological situations such as free viewing has never been demonstrated.

**Fig. 1:**
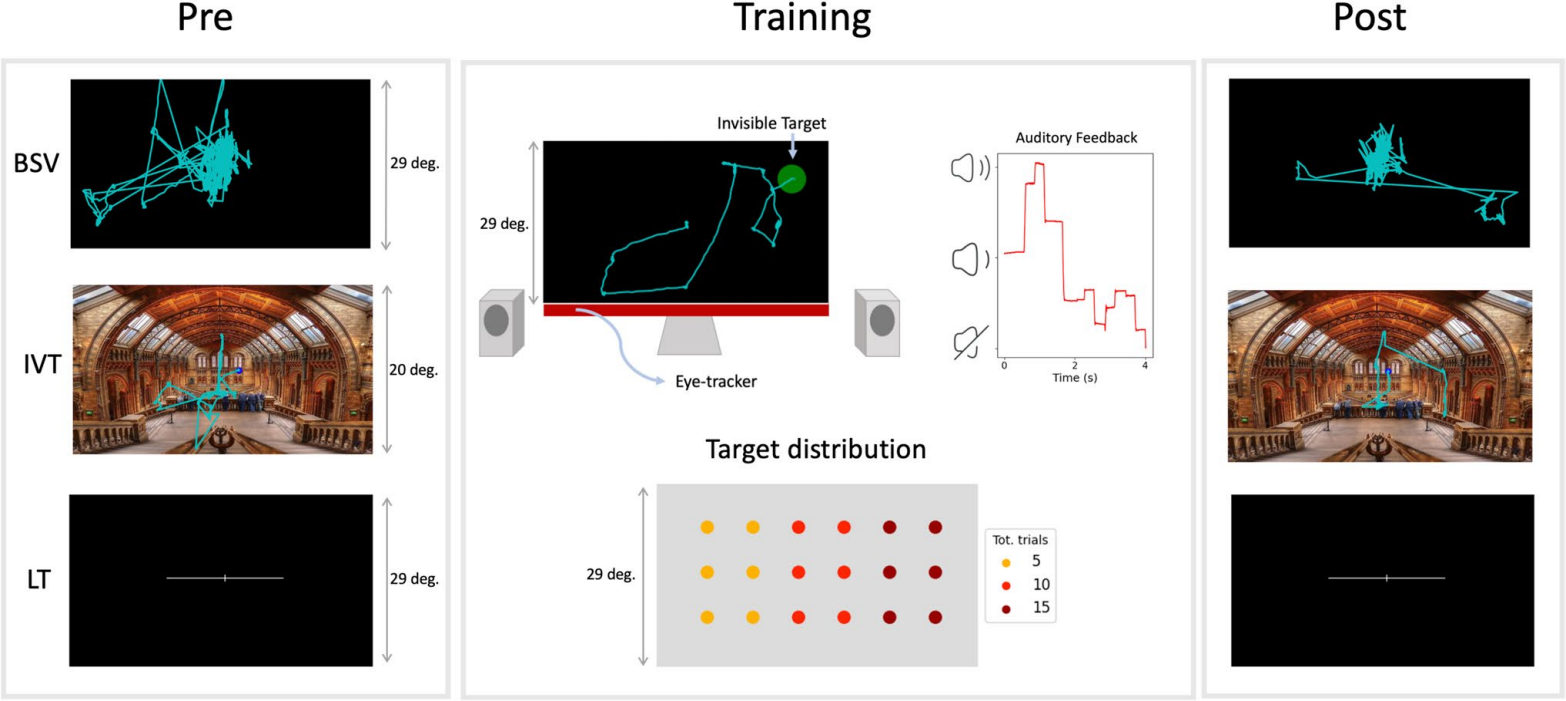
Experimental paradigm. Pre- and post-training assessment is illustrated in the left and right columns, respectively. BSV = blank screen viewing, IVT = image viewing task, LT = landmark task. The light blue-colored lines superimposed on the images in BSV and IVT represent the gaze scan paths from one participant in the right bias group. The central panel illustrates the training phase, with the top images showing the participant’s scan path towards the invisible target and the corresponding lowering of the auditory feedback volume in one trial of the AVS task. The bottom image shows the spatial distribution of targets on the screen (for the right bias condition), with colors indicating the total number of trials for each target position

In this pre-registered study (https://osf.io/ws5dx/), we examined the transfer of spatial bias acquired during a novel closed-loop Audio-Visual Search (AVS) task to other unrelated visuospatial tasks. Participants had to move their gaze (monitored through an eye-tracker) across the computer screen to search for an invisible target location, which was more likely to occur in one of the two hemifields. Auditory feedback provided a continuous, real-time signal of the motor error, that is the distance between gaze and target in eye-centered coordinates (Krauzlis et al., 1997), encoded using sound loudness (Fig. 1). Note that auditory feedback is commonly used to convey spatial information in various assistive technologies (e.g., parking aids; visual impairment assistance) and gaze-contingent auditory feedback has been proposed as a means to facilitate users’ search of visual targets in the context of human-computer interaction studies (Losing et al., 2014). However, the targets remain invisible in our paradigm, thereby engaging an abstract level of spatial representation detached from perceptual objects.

Observers were divided into two groups, respectively performing the AVS task with target probability biased either to the right or the left hemifield. We assessed the transfer of statistical learning using three visuospatial tasks that were performed both before and after the AVS task: (1) a blank screen viewing (BSV) (“resting”) condition, requiring participants to simply view the blank computer screen; (2) an image viewing task (IVT), in which observers looked at natural images in a free-viewing paradigm; and (3) a landmark task (LT), where participants had to judge which of the two ends (left vs. right) of a pre-bisected horizontal line was longer. The BSV condition captures spontaneous attentional biases and intrinsic eye-movement dynamics in the absence of external stimulation (e.g., Zangrossi et al., 2021); the IVT tests exploration biases in natural settings, which require more complex oculomotor exploration (Nuthmann & Matthias, 2014); the LT is sensitive to (allocentric) attentional biases, and has been widely exploited for the evaluation of attentional disorders or to assess the effect of rehabilitation procedures (e.g., prismatic adaptation; Schintu et al., 2014). These tasks enabled assessment of both overt and covert attentional biases, and therefore a more comprehensive evaluation of the transfer.

Regarding the AVS task, we tested the following (pre-registered) predictions: (1) a systematic decrease in the distance between target and gaze over time within each trial, indicating that the auditory biofeedback effectively guided visual search, as well as a decrease in the target-gaze distance across trials, indicating a task learning effect; (2) a more efficient search for targets in the biased hemifield (i.e., a faster decrease in gaze-target distance) as participants progress in the task, indexing statistical learning.

Regarding the transfer of statistical learning from AVS task to the other spatial tasks, we tested the following (pre-registered) directional hypotheses. For BSV, a horizontal shift of fixation location (i.e., barycenter, which corresponds to the center of mass) towards the biased hemifield at post-test compared to the pre-test assessment, indicating transfer of bias from AVS to free viewing. For IVT, a coherent shift of fixation location towards the biased hemifield compared to the pre-test assessment, indicating transfer of bias from AVS to visual exploration of natural images. For LT, a change in the Point of Subjective Equality (PSE) towards the biased hemifield at post-test compared to the pre-test assessment.

Finally, in exploratory analyses, we investigated the eye-movement dynamics during free viewing at pre-test to assess the possible influence of “viewing styles” (Zangrossi et al., 2021) and natural spatial biases (Bowers & Heilman, 1980) on AVS performance and transfer of statistical learning. Recent studies have shown that observers visually explore natural images using different viewing styles: some (“static”) individuals show long fixations and large gaze steps, while other (“dynamic”) individuals show short fixations and smaller gaze steps (Zangrossi et al., 2021). These styles are associated with differences in resting EEG signals (Celli et al., 2022). Moreover, observers show natural biases in visuospatial attention, typically towards the left side (Bowers & Heilman, 1980; Jewell & McCourt, 2000; Nuthmann & Matthias, 2014), related to neuroanatomical asymmetries of the attentional system (Corbetta & Shulman, 2011) and/or culture (Rinaldi et al., 2014). Entraining novel spatial biases through statistical learning may also have some clinical applications in counteracting lesion-induced biases as in stroke (Blini et al., 2016; Bonato et al., 2010). Statistical learning of location probability is preserved in patients with visuospatial neglect (Geng & Behrmann, 2002) and presenting targets in the neglected hemispace may have the potential to ameliorate visuospatial deficits (Shaqiri et al., 2013). For these reasons, the demonstration that statistical learning transfers from our AVS task to other spatial tasks would be highly significant (beyond its theoretical value) for clinical applications.

## Method

### Sample

A priori statistical power analysis was conducted, based on the results of a preliminary study (N = 24) with the same task and asymmetric target distribution. Power of an effect size equal to the preliminary evidence (Cohen’s d = 0.4) was assessed. A design based on one-tailed paired t-test comparisons was considered, as most of the study’s hypotheses are directional, and used an alpha level of 0.05. The analysis suggested that with an effect size of 0.4, 92% statistical power would be achieved after enrolling 60 participants. We planned an interim analysis with 40 participants (estimated power: 80%) to optimize the sampling plan, and the p-value for accepting H1 was adjusted accordingly to 0.0329 for both the interim and final analyses (Pocock, 1977), to ensure a fixed type-I error rate. Since the assessment of statistical learning transfer was the primary goal of the study, we planned the possibility of early stop at the interim analysis if the main effect of statistical learning (one-sided, coherent shift) was confirmed in at least two of the three follow up assessment tasks.

Participants were recruited through advertisements among university students at the University of Padova, Italy. Inclusion criteria were age between 18 and 40 years, having no psychiatric or neurological condition, and having normal or corrected-to-normal vision. The recruitment stopped after performing the interim analyses on N = 40. The mean age of the sample was 23.8 years, with a total of 23 women recruited. Regarding handedness, only two participants reported being left-handed. Written informed consent was obtained from all participants, and the study was approved by the University of Padova psychological research ethics committee (prot. N° 4218) and adhered to the standards provided by the Declaration of Helsinki.

### Apparatus

The study employed a computer running on Windows 10, a remote infrared-based eye-tracker (TOBII Spectrum) with a sampling rate of 600 Hz, and a 23.8-in. monitor with a 1,920 x 1,080 pixels resolution. A chinrest was placed in front of the eye-tracker, distanced 57 cm, allowing participants to maintain a stable position throughout the session. All tasks were implemented using custom Python code within the open-source program OpenSesame (Mathôt et al., 2012) running with Python 2.7. To provide the auditory biofeedback, loudspeakers were placed on both sides of the eye-tracker.

### Stimuli

#### AVS

Participants were tested using an audio-visual search task on a black screen overlaid with a 10 × 18 grid of small white dots that provided visual landmarks. At trial start, a white fixation dot appeared to ensure gaze stability, with trials beginning as soon as fixation was kept at the center of the screen for 0.5 s. The invisible targets had a radius of 4.1°, chosen for optimal task sensitivity and difficulty through piloting. Real-time auditory feedback was computed based on the distance between gaze position (sampled at 600 Hz) and target (i.e., motor error). The feedback sound (440 Hz) was exponentially mapped to volume (up to a maximum distance of 17.8°) to accommodate for logarithmic intensity perception. Silence indicated that the gaze was on target. Trials ended when the target was fixated for 500 ms, indicating a successful trial, or when reaching the deadline of 10 s. The targets could appear along six different horizontal positions (from the center in both direction: 3.8, 11.4, 19.9°), and three vertical positions (from the center in both directions: 0, 7.5°), thus describing a matrix structure with a total of 18 unique target positions (Fig. 1). The total number of trials was 180, with a distribution of targets from left to right which was 90 - 60 - 30 trials for the left bias group, or 30 - 60 - 90 trials for the right bias group. The trial order was randomized across participants.

#### BSV

In this condition the screen was black and empty, with no visual stimuli presented. The resting condition lasted for 1 min. Participants were instructed that they could move their eyes freely as long as they remained within the limits of the screen.

#### IVT

For the image viewing task we selected pictures used in a previous study conducted by Zangrossi et al. (2021), where they studied the dynamics of free visual exploration of 185 pictures in a sample of 114 healthy participants. Based on the available fixation density maps, we chose 30 images with a “balanced exploration” of the left and right part of the image using the following procedure: first, the horizontal center of mass for each image was calculated using the corresponding fixation density map. Next, a subset of stimuli was selected such that the distribution of their centers of mass closely aligned with the horizontal midpoint of the stimuli. This method ensured that, while individual stimuli might encourage leftward or rightward exploration, the overall distribution across the 30 selected images would likely result in a balanced exploration centered around the screen's midpoint.

Each image was presented for 5 s, with an inter-trial-interval of 1.5 s characterized by an empty black screen. The image size (width 27°, height 20°) reflected the proportions used in Zangrossi and colleagues’ study. All images were photos of real scenes depicting indoor and outdoor landscapes, with or without humans. The order of image presentation was fixed, and the set was repeated in the pre and post assessment with the rationale that a within-image comparison would provide greater sensitivity to attentional biases. Although repeated image exposure can alter eye-movement patterns, Kaspar and Koenig (2011a, b) demonstrated that individuals revisiting the same image for a second time tend to exhibit longer fixation durations and smaller saccades but continue exploring similar regions and remain attracted to salient features. Based on these findings, repeated image presentation seems an adequate methodological choice for studying attentional biases since exploration patterns tend to be rather reliable.

#### LT

For the landmark task, the stimulus was a white horizontal line (width 19.2°, height 0.08°) bisected by a white vertical line (width 0.08°, height 0.5°) presented on a black screen. The horizontal line was bisected in one of 11 different and equidistant positions (total range of +/- 0.68° from the veridical center), including the veridical center. Each unique bisection position was repeated eight times, totaling 88 trials. The trial order was randomized across participants. The start of a new trial was preceded by a white visual noise mask presented for 1 s to minimize the retinotopic trace of the previous stimulus.

All assessment tasks entailed no fixation dot, to prevent repetitive reorienting to the center of the screen which could in turn mask any learning-related attentional bias.

### Procedure

Participants were tested in a dark room, seated on a chair and with the head resting on a chin-and-forehead rest, to restrict all head movements. Before starting the experiment, we calibrated the eye tracker through a nine-point procedure. Written instructions were given in each task and six practice trials were given at the beginning of the AVS task (later discarded). Based on shuffle randomization (i.e., shuffling of an ordered list with balanced groups assignment), participants were assigned to the right bias group or to the left bias group. As an interim analysis was planned, shuffle randomization was restricted initially at the first 40 participants to obtain balanced groups. The experiment was divided into three parts, namely pre-assessment, training phase and post-assessment (Fig. 1). During the pre-assessment we administered, in a fixed order, the BSV lasting 1 min (black screen, no stimuli), IVT and LT. In the post-assessment, we repeated the tasks of the pre-assessment phase, with the same order. For the BSV the participants were instructed to rest without fixating outside of the monitor. For the IVT, participants were simply instructed to look at the presented images. Lastly, for the LT, participants were instructed to judge whether the vertical line bisecting the horizontal line was closer to the right or left end of the latter by pressing respectively the right (right index finger) or left keyboard arrow (left index finger). The trial was terminated by the button-press response or by a timeout of 5 s. In the training phase, we presented the participants with the AVS with targets asymmetrically distributed towards one hemispace, depending on which group they were assigned to. Finally, we assessed explicit awareness of the biased hemifield with an open question at the end of the experiment. Participants who explicitly mentioned an asymmetric target distribution, with more targets in the biased hemifield, were labeled as “explicit” learners. Debriefing was done once the experiment was concluded.

### Data preprocessing

During each task, the position of left and right eyes was recorded. When data points were missing (e.g., due to blinks), they were estimated using linear interpolation to fill the gaps. However, if a trial had more than 40% of its eye-tracking data missing in a continuous sequence, it was considered unreliable (invalid) and excluded from the eye-movement analysis. For both BSV and IVT, saccades were estimated based on velocity distribution with the “saccades” R library, according to Engbert and Kliegl’s (2003) algorithm, and further filtered following Hooge et al. (2022) selection criteria. Details about the preprocessing steps are reported in the Supplemental Information (SI).

### Statistical analyses

Analyses are divided in pre-registered and exploratory, as outlined in the pre-registration recorded in the Open Science Framework website (https://osf.io/ws5dx/). All analyses and plots were performed with R (R Core Team, 2024) and Python 3 using *glmmTMB*, *saccades*, *ggplot2* and *Seaborn* packages (Brooks et al., 2017; von der Malsburg, 2015; Waskom, 2021; Wickham et al., 2016).

#### Pre-registered

##### AVS task

To assess the efficiency of the AVS task, a generalized mixed-effects model (GLMM) was fitted. The outcome variable was the Euclidean distance between the registered gaze and actual target. The measurements were binned with the median value within 500-ms intervals. The conditional distribution of the response variable was a gamma distribution with a log link function to account for the positive skewness of the outcome. The predictors were Bias (categorical variable with two levels, whether the target was in the biased or unbiased hemifield), Trial Number (continuous variable, trial range 1–180), Time On Trial (continuous variable, time range of 0–10 s with evenly spaced intervals of 0.5 s), as well as all their interactions. Continuous variables were mean centered and scaled to unit variance (i.e., standardized). Participants were included as a random grouping factor with random intercepts and random slopes for each fixed effect. Maximum likelihood was used for estimating model parameters and likelihood-ratio tests for assessing fixed effects statistical significance.

##### Transfer tasks

To assess the after-effects of the biased visual search, three one-sample t-tests (one-sided) were conducted on pre versus post differences in the transfer tasks. To collapse both bias groups to a common shift measure, individual changes in the left bias group were reversed in sign.

Specifically, for both BSV and IVT, we computed the mean horizontal fixation position in pre- and post-test, then subtracted the two measures to index the shift for each participant. For the IVT, this resulted in 30 shifts per participant (one per image), which were then collapsed with the median. The median was used because nine participants showed non-normal distributions (according to D’Agostino – Pearson Omnibus test), which results in the mean (pre-registered statistic) being an inadequate centrality measure (further details and robustness check are reported in the SI, Table S1).

Concerning the LT, PSEs for pre- and post-test were estimated for each participant by fitting a logistic regression using the *quickpsy* library (Linares & López-Moliner, 2016) and then subtracted to obtain a difference measure.

#### Exploratory

To assess the robustness of our findings on the transfer of statistical learning to the IVT, we carried out supplementary analyses suggested during the review process. First, we repeated the same pre-registered analysis excluding all images containing humans to assess the possible impact of social content that is known to attract attention (Yarbus, 1967). Second, we examined whether the induced attentional bias was already manifest in the early phase of visual exploration. We considered the saccades in the first second from trial onset and computed the median horizontal shift for each image. We then repeated the pre-registered IVT analysis on this new index. Third, we assessed the correlation between shifts in barycenter observed during IVT and BSV to examine the within-participants consistency.

We also carried out a set of exploratory analyses (anticipated in the pre-registration report) aimed at investigating whether individual differences in eye-movement dynamics (Zangrossi et al., 2021) modulate the performance in the AVS as well as the changes observed across pre- and post-assessment phases. This would have important translational implications, as it would allow one to forecast who might benefit more from the training. Indeed, eye movements have a low latent dimensionality (Poynter et al., 2013; Zangrossi et al., 2021), with different emerging viewing styles (in particular, statics vs. dynamic viewers; see Zangrossi et al., 2021) that could explain variability both in visual search performance and statistical learning and that refer to stable properties of spontaneous brain activity (Celli et al., 2022). To evaluate this hypothesis, we conducted an analysis on eye-movement statistics during free viewing similar to the one reported by Zangrossi et al. (2021). We extracted from the pre-BSV and pre-IVT 12 features describing spatiotemporal characteristics of fixation and eye-movement steps (detailed in the SI). The features selected were a subset of a larger pool of eye-movement indices extracted by Zangrossi and colleagues, and were based on fixation properties, the rate of direction flips (i.e., movement inversion) in successive eye samples (here termed gaze flips, specified in the horizontal and vertical axis), and the Euclidean distances between successive eye samples (here named gaze step). Next, these 12 indices were reduced, separately for each task, via Principal Component Analysis (PCA). The first three Principal Components (PCs), which explained a large proportion of the variance (> 78%), were used as potential predictors of AVS performance and the statistical learning transfer effect, as outlined below. In the pre-IVT the first PC loaded on large gaze steps and long fixations, the second PC weighted on small gaze steps and long fixations, while the third PC loaded on brief fixations in the first second of image exploration and fewer gaze flips in general. In contrast, in the pre-BSV the first PC loaded on short gaze steps and long fixations, the second PC weighted on brief and numerous fixations and fewer gaze flips while the third PC loaded on long fixations in the last 15 s of BSV and in general briefer total fixation duration.

Furthermore, we aimed to explore the role of the natural spatial bias, which is a common leftward bias present in the neurologically healthy population (Bowers & Heilman, 1980), as a moderator of statistical learning transfer. Accordingly, we computed the median horizontal position of fixations recorded within the first second of pre-BSV and pre-IVT. This index was chosen based on previous studies which have shown the largest leftward deviation of fixations in the first second of exploration of various images (Chiffi et al., 2021; Nuthmann & Matthias, 2014; Ossandón et al., 2014). Next, to disentangle the relation between pre-existing biases and transfer, we took the absolute values of the computed index and marked it as a positive value if the participant exhibited the same bias as the experimental manipulation (e.g., rightward natural bias and right bias group), or as a negative value if the participant showed opposite biases. This index is here referred to as “congruent bias.”

Three multiple linear regressions were built to explain the main outcomes of the study. Specifically, the AVS mean visual search time was regressed on the first three PCs of pre-BSV and pre-IVT. Moreover, the change in the horizontal fixation’s barycenter observed between pre- and post-BSV was regressed on the first three pre-BSV PCs and the pre-BSV congruent bias. In the same manner, the change in the fixation’s barycenter observed between pre- and post-IVT was regressed on the first three pre-IVT PCs and the pre-IVT congruent bias.

## Results

Regarding participants’ performance in the AVS task, targets were found on average in 86% of all trials (*SD* = 12%). The grand mean elapsed time to complete the trial was *M* = 5 s, with *SD* = 1.1 s. The target location manipulation, i.e., higher frequency of certain locations over others, remained unnoticed in 38 of 40 participants.

### Pre-registered

#### AVS task

**Fig. 2:**
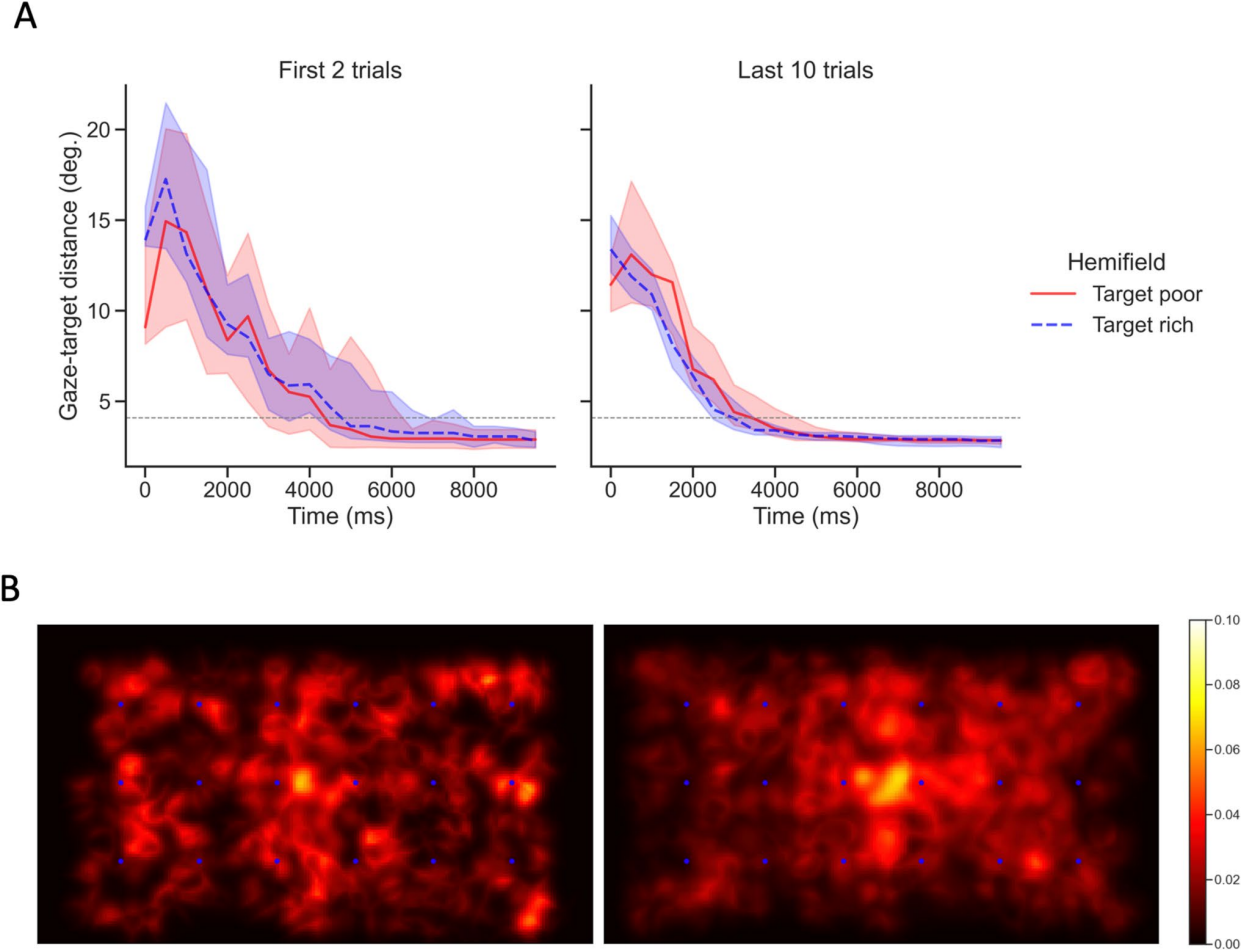
Development of the attentional bias in time and space during the AVS task. **A.** Gaze-target distance is plotted as a function of time on trial in the target rich (broken blue line) and target poor (solid red line) hemifields. The lines represent, at each time bin of 500 ms, the median distance across participants with the bands highlighting the 95% confidence intervals. The horizontal dotted line represents the minimum distance threshold for reaching the allocated target. The left panel represents the median across the first two trials, whereas the right panel is the median across the last 10 trials. For this plot, once the target has been successfully reached by the participant, missing points in the time series have been set to the last recorded distance measure. **B.** Fixation density maps showing the attentional allocation in the first two (left panel) and last 10 trials (right panel). To highlight the learned bias across hemifields, targets and gaze positions of the left bias group have been flipped onto the right hemifield. Each map is based on the median of six fixation density maps, calculated for the six unique horizontal target positions. To reduce the strong influence of central fixation at the start, the first 750 ms of data were excluded before computing these maps. The superimposed blue dots represent the target positions, whereas the color scale represents the fixation density (i.e., probability)

As predicted in the first hypothesis, the likelihood-ratio test of the fitted GLMM (Table 1) indicated a main negative effect of Time On Trial and Trial Number on gaze-target distance. Moreover, all two-way interactions between the predictors were statistically significant. Indeed, as depicted in Fig. 2A, participants decreased their distance from the target as a function of the Time On Trial, particularly in the last trials of the AVS, reflecting the learning effect (significant interaction between Time On Trial and Trial Number). Furthermore, in line with our second hypothesis, the effect of Bias unfolded as a function of Trial Number and was modulated by Time On Trial. The development of the spatial bias can also be visually appreciated in the Fixation Density Maps obtained in the initial and final trials of the AVS (Fig. 2B). Overall, the largest influence on gaze-target distance was driven by Time On Trial, with a 0.81 multiplicative decrease of the gaze-target distance every 2.5 s. (i.e., one standard deviation of the Time on Trial variable).

**Table 1:** GLMM with gaze-target distance as outcome variable and Time On Trial, Bias, Trial Number, and their interactions as fixed effects. Estimates are multiplicative factor change in the gaze-target distance for one unit change in the predictors (continuous variables are standardized)

Fixed effects	Estimate	Chi-square	*P* value
Time On Trial	0.81	82.88	<.001
Trial Number	0.96	17.51	<.001
Bias	1.01	2.10	.147
Time On Trial : Trial Number	0.99	30.94	<.001
Time On Trial : Bias	1.01	4.33	.037
Trial Number : Bias	1.01	5.56	.018
Time On Trial : Trial Number : Bias	1.00	2.47	.116

#### Transfer tasks

**Fig. 3:**
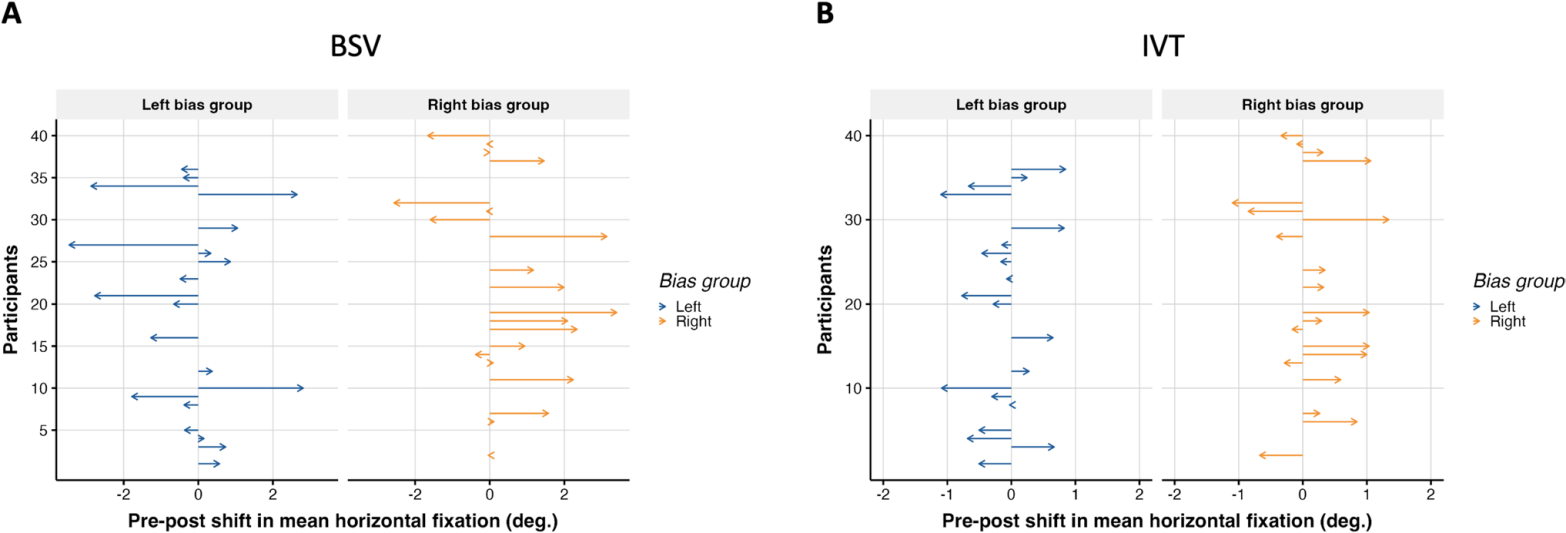
Post-training free-viewing barycenter shift.** A.** For each participant (Y axis) the arrows depict the change in the mean horizontal fixation between post-BSV minus pre-BSV (X axis). Participants are divided in the left and right bias group. Participants’ numbering was attributed during recruitment and irrespective of group. **B.** For each participant (Y axis) the arrows depict the change in the mean horizontal fixation between post-IVT minus pre-IVT of all paired images (X axis). The changes for each image are collapsed using the median value. Participants are divided in the left and right bias group

There was a coherent change in the mean fixations position in the horizontal axis towards the biased hemifield, both at rest (BSV: *M* = 0.49°, *SD* = 1.61, 95% *CI* [0.05, inf], *t*(39) = 1.91, *p* =.031), and during image viewing (IVT: *M* = 0.2°, *SD* = 0.64, 95% *CI* [0.02, inf], *t*(39) = 1.92, *p* =.03). Both effects sizes were small, with a Cohen’s d of 0.3. Regarding BSV, participants exhibited similar oculomotor behavior in the pre- and post-assessment phases, namely long-lasting fixations (pre-assessment *M* = 2.1 s; post-assessment *M* = 2.0 s), and saccades spanning around 6° (pre-assessment *M* = 6.8°; post-assessment *M* = 6.7°). Individual shifts of each participant are depicted in Fig. 3. For the LT, in contrast to the initial hypothesis, there was no statistically significant change (*t*(39) = − 1.24, *p* =.889).

### Exploratory

#### Supplementary analyses of the IVT

These analyses (suggested during the review process) aimed at assessing the robustness of our findings on the transfer of statistical learning to the IVT. First, we repeated the pre-registered analysis excluding all images containing humans, considering the finding that gaze is attracted toward social content (Yarbus, 1967). The results on the subset of images (N = 12) replicated those obtained on the full set (*t*(39) = 2.02, *p* =.024), suggesting that the degree of transfer was similar across types of images, regardless of the presence of social content. Second, we examined whether the bias was present in the earliest phase of visual exploration. We therefore repeated the pre-registered analysis on the horizontal shift computed from the saccades executed during the first second from image onset. The analysis confirmed the post-training attraction toward the biased hemifield (*t*(39) = 1.91, *p* =.031). This finding extends the pre-registered results and indicates that the developed bias affected the barycenter as well as the horizontal direction of the first saccades. Finally, we examined the correlation between the post-training bias in the IVT and the BSV. The Pearson correlation was very weak (r = 0.1), indicating that the transfer effects on the two conditions varied considerably within-participants. Nevertheless, the probability of alignment among bias direction (left vs. right) in AVS bias condition, IVT post-training bias and BSV post-training bias was 0.45. This probability was significantly higher than the chance level of 0.25 (binomial test, p =.004).

#### Relation between free-viewing style and training outcomes

**Fig. 4:**
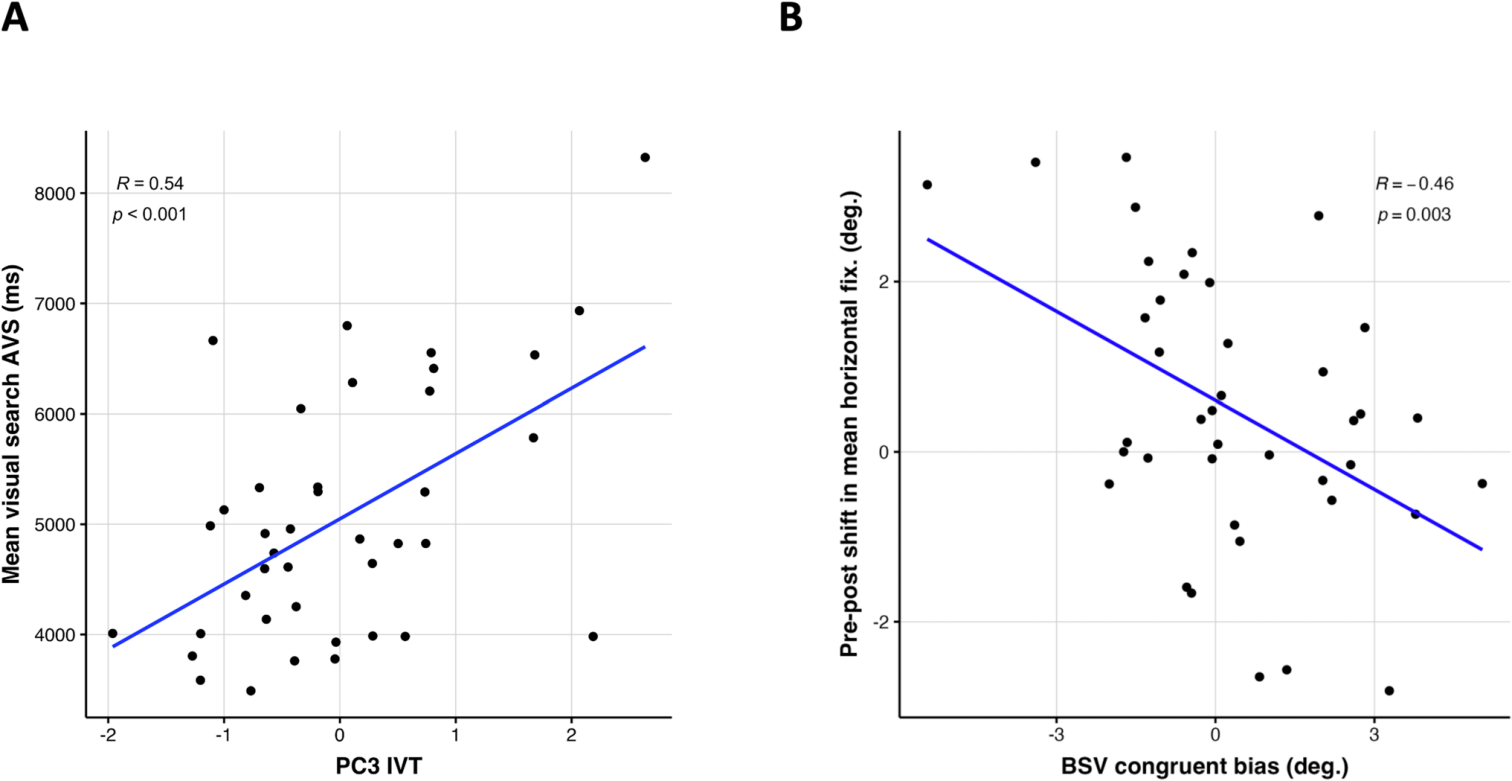
Correlation between free viewing’s characteristics and AVS performance and bias transfer.** A.** The figure represents the AVS mean visual search time as a function of the PC3 IVT scores, which are loaded on brief fixations and frequent gaze flips. **B.** The figure shows the pre- to post-assessment shift in the mean horizontal fixation observed during BSV (positive values indicate a shift toward the biased hemifield) as a function of the congruent bias index computed in the first second of pre-BSV

These exploratory analyses investigated whether the training outcomes were influenced by individual characteristics such as viewing style. Principal components (PCs) of eye-movement statistics during pre-BSV and pre-IVT are summarized in SI Appendix, Fig. S1 and Fig. S2. The first three PCs of BSV and IVT explained respectively a total of 79% and 85% of the variance. The model predicting the AVS mean visual search time revealed only one significant predictor, namely the IVT PC3 (*t*(33) = 2.88, *p* =.006). Thus, participants with brief fixation duration during the first quarter (i.e., 0–1.25 s) of image exploration and fewer gaze flips showed afterwards longer search time during the AVS (Fig. 4A).

Concerning the fixation’s barycenter shift, the only significant predictor was the congruent bias for both IVT (*t*(35) = − 2.18, *p* =.035) and BSV (*t*(35) = − 2.94, *p* =.005) models. Hence, participants with a congruent bias during free viewing and experimental group assignment were more likely to show smaller or even reversed fixation shift (Fig. 4B).

## Discussion

There are three main findings in this study based on the pre-registered hypotheses. Firstly, participants learned to localize accurately a "hidden" target in the visual field by moving their eyes based on an auditory feedback signal. Secondly, they learned the probability of target location distribution in the visual field even in the absence of awareness. Thirdly, this form of location probability learning generalized to free-viewing behavior, both at rest (i.e., looking at a blank screen) and while viewing natural images. There were other interesting exploratory results. First, we confirmed the low dimensional latent structure of eye-movement features across participants during free visual exploration, and we found that one of the components describing free image viewing was related to performance in the audiovisual search task. A second result of interest is that participants with a strong natural bias in the same direction as the bias induced during AVS showed a diminished or even reversed post-training bias.

Participants showed good performance in the novel AVS task and found 85% of the invisible targets within 5 s. Learning effects were highlighted by the decreasing error (i.e., gaze-target distance) across trials and, most importantly, with a larger effect in the target-rich hemifield. This aligns with prior findings suggesting that people are more likely to direct their initial gaze toward areas where targets are frequently found (Jiang et al., 2014; Salovich et al., 2018). However, our task significantly departs from classic probability cueing paradigms. Most notably, targets were invisible (i.e., there was no visual cue) and were only represented by the auditory feedback conveying gaze-target distance in eye-centered coordinates. Thus, the sensory modality initiating and driving the process was auditory, and visual exploration was driven by top-down signals. Moreover, the learning phase was considerably shorter than in other studies (Jiang et al., 2015; Salovich et al., 2018). Notably, the learning of regularities during the AVS remained unnoticed in all but two participants. Nonetheless, explicit awareness of target distribution has been shown to have no effect on the developed bias (Jiang et al., 2018).

The attentional bias induced by the AVS task significantly influenced subsequent free viewing. The average gaze barycenter shifted towards areas that were previously target-rich. Despite the significant effects at the group level, the horizontal shift in the barycenter showed considerable within-participant variability across BSV and IVT. Nonetheless, the concordance probability between bias direction in the IVT and BSV, conditioned on AVS bias condition, was significantly higher than chance level. The cause of this discrepancy remains to be investigated. However, to the best of our knowledge, this is the first study demonstrating transfer of location probability learning to free viewing (both at rest and with natural images), which has no relation to the task eliciting statistical learning and is the closest to an ecological setting. Indeed, previous work has shown transfer of location probability learning to related visual search tasks (Jiang et al., 2015; Salovich et al., 2018) but not to other tasks such as treasure-hunt (Jiang et al., 2015), scene search (Addleman et al., 2019), image search (Sha et al., 2018), and scene memory (Addleman et al., 2018). In contrast to free viewing, there was no evidence of transfer to spatial judgments in the LT task. This discrepancy can be attributed to the preferential recruitment of different cognitive processes: the AVS engaged spatial selection while the LT required to judge spatial extent, which respectively exploit egocentric (Jiang & Swallow, 2013) vs. allocentric frames of reference. These findings align with another study where exploration biases, elicited by a gaze-contingent task, influenced viewing patterns in a line bisection task but not the actual responses (Foulsham et al., 2013). It is also worth noting that spatial biases in perceptual judgments can arise from both object-centered and space-based reference frames (Orr & Nicholls, 2005) and can be modulated by spatial cueing (Nicholls & Roberts, 2002), thereby suggesting complex interactions that might hinder the possibility to observe a transfer effect. Alternatively, the lack of transfer could be simply explained by a rapid decline of the attentional bias over the time course of the post-assessment phase. That is, the attentional bias may have been too weak to influence the LT because the latter was always the last task in our protocol.

We highlight that the AVS task engaged an abstract level of spatial representation detached from perceptual objects, in contrast to classic paradigms used to investigate location probability learning (or visual perceptual learning, Lu & Dosher, 2022). This feature might have favored transfer to free viewing. Indeed, it is widely assumed that we sample visual information according to an ever-changing spatial priority map, which is shaped by selection history in addition to bottom-up and top-down mechanisms (Theeuwes, 2019). The lack of explicit goals in our free-viewing conditions (and even of sensory stimuli in the rest condition) might have favored selection history as a modulator of the priority map, thereby unveiling the transfer of statistical learning. This significantly extends previous findings, and it is consistent with the hypothesis that our history of visual selections significantly shapes where we direct our attention in the visual world (Awh et al., 2012; Jiang, 2018; Theeuwes, 2019). Our results challenge the task-specificity of statistical learning and solicit further investigations of more ecological and constraints-free scenarios.

Our supplementary analyses of the IVT revealed that the transfer was robust across image content and that the bias towards the target-rich hemifield was present in the earliest phase of free image viewing. The latter finding suggests that the attentional bias developed through statistical learning acts as a prior that influences visual selection despite the well-established role of visual saliency and semantics in driving the initial image exploration (Henderson & Hayes, 2017). Future studies could explore in more detail the temporal dynamics of these biases. Finally, we investigated individual differences in baseline free-viewing behavior to assess if they could predict AVS task performance and learning transfer. The largest variation (i.e., PC) in exploration dynamics during image viewing distinguished participants with long fixations and large gaze shifts from the opposite pattern, a result resemblant of the static and dynamic styles found by Zangrossi et al. (2021). Intriguingly, the way participants initially explored an image predicted their performance in the AVS task, with those who fixated the image more rapidly generally taking longer to find the target. We speculate that a slow-paced visual sampling early on during visual exploration may underpin a more efficient processing of the auditory feedback and subsequently a more accurate reorienting of attention. This result might be useful to forecast who would be more successful in the training, laying empirical ground for the translation of the AVS in clinical settings.

While free-viewing patterns explained search performance, the degree of transfer was instead predicted by natural spatial biases. The presence and strength of individual bias at baseline, that is the natural tendency to deviate the gaze leftward or rightward during the first second (Chiffi et al., 2021; Nuthmann & Matthias, 2014; Ossandón et al., 2014), was found to modulate transfer of statistical learning. This result can be interpreted in terms of a limit to how much our natural biases can be further magnified by statistical learning: a strong initial bias leaves less room for change in the same direction. A similar finding has been observed with prism adaptation, where pre-existing (i.e., baseline) spatial biases were shown to constrain post-adaptation effects (Goedert et al., 2010).

A limitation of the present study is the focus on an overt attentional marker of statistical learning, namely the change in the average fixation barycenter during free viewing. However, the variability of transfer and the interaction with pre-existing biases highlights the need for future research to obtain a finer-grained view of free-viewing behavior to assess more subtle changes, such as saccade-related statistics and entropy-based indexes, and most importantly the maintenance over an extended time window.

In conclusion, the study highlights the potential of a novel gaze-driven auditory biofeedback system for visual search without visual cues. Learning of target locations during AVS was effectively transferred to free-viewing behavior, as shown by a change in gaze barycenter. This challenges the view of task specificity of location probability learning and urges future work to extend investigations to ecological and unconstrained tasks. Exploratory findings suggest that individual differences in free-viewing patterns and pre-existing spatial biases respectively modulate AVS performance and statistical learning transfer. Beyond the theoretical implications, our results serve as a proof of concept for a clinical application to patients with pathological spatial biases. The protocol could leverage on the biofeedback system to guide the gaze in an online closed-loop fashion and on the manipulation of target frequency to shape an attentional gradient to counteract the pathological spatial bias.

## Supplementary Information

Below is the link to the electronic supplementary material.

Supplementary file1 (PDF 326 KB)

## Data Availability

The datasets generated and analysed during the current study are available in the OSF repository, https://osf.io/ws5dx/.

## Code availability

Code is publicly available in the OSF repository, https://osf.io/ws5dx/.
