# Supplementary material for: Spatial regularities in a closed-loop audiovisual search task bias subsequent free-viewing behavior
Source: Psychon Bull Rev. 2025 Jul 7;32(6):2977–89. doi: 10.3758/s13423-025-02703-8 (PMC12627162; doi:10.3758/s13423-025-02703-8)
Supplement: Supplementary file 1 — Supplementary file1 (PDF 326 KB) [file 13423_2025_2703_MOESM1_ESM.pdf]

Supporting Information for

Spatial regularities in a closed-loop audiovisual search task bias subsequent free viewing behavior

Sebastiano Cinetto, Elvio Blini, Andrea Zangrossi, Maurizio Corbetta, Marco Zorzi

Corresponding author: Marco Zorzi
Email: marco.zorzi@unipd.it

Methods

Data Preprocessing

For FV, IVT and LT the following criteria concerning invalid trials were respected.
FV. Any participant presenting in the FV, either at pre- or post-assessment, an invalid trial would have been discarded from the analyses concerning this task. There were no invalid trials, thus all participants were kept for the analyses concerning FV.
IVT. Any participant with more than 40% of invalid trials across all repeated images in the pre- or post-assessment would have been excluded from the analysis concerning this task. The invalid trials were on average M = 0.85% with SD = 2.6%: no participant was removed.
LT. Any participant failing to respond in more than 20% of all trials would have been discarded from the LT analyses. All participants provided more than 80% of all required responses.
To accommodate the different task nature of FV and IVT saccades were identified if they were respectively 9 and 6 standard deviations above the median velocity of all eye samples recorded for each participant. Moreover, to ensure robust computation, a posteriori selection rule for saccades and fixations was followed, as suggested by Hooge et al. (2022). In a first step, saccades with either amplitude lower than 1.0 deg., or duration lower than 10 ms were removed. Fixations interleaved by invalid saccades were merged with their mean position, weighted by their durations. In a second step, fixations with duration lower than 80 ms were discarded. Critically, in the FV there was a considerable number of fixations gauged as artifacts, likely due to the long time spent resting without visual stimuli. Three types were noted and removed: fixations with extremely low or high spatial dispersion, as warned by the "saccades" library (respectively mirroring blinks and eye-tracker measurement errors), and fixations recorded outside the screen frame.

Eye movement features and PCA

The features extracted from the gaze data and included in the PCA were: the mean and standard deviation of fixation duration; the mean fixation duration in the first and fourth quarter of exploration time; the total time spent during fixations (i.e., total fixation duration); the total number of fixations; the rate of horizontal (X) and vertical (Y) gaze flips observed in the eye-samples (i.e., how many times the eyes move in opposite direction compared to previous movement in the horizontal or vertical axis in the given trial); the mean and standard deviation of the Euclidean distance between each successive eye sample (i.e., gaze step) observed in the whole trial, and in the first and fourth quarter of exploration time.

Figures and Tables

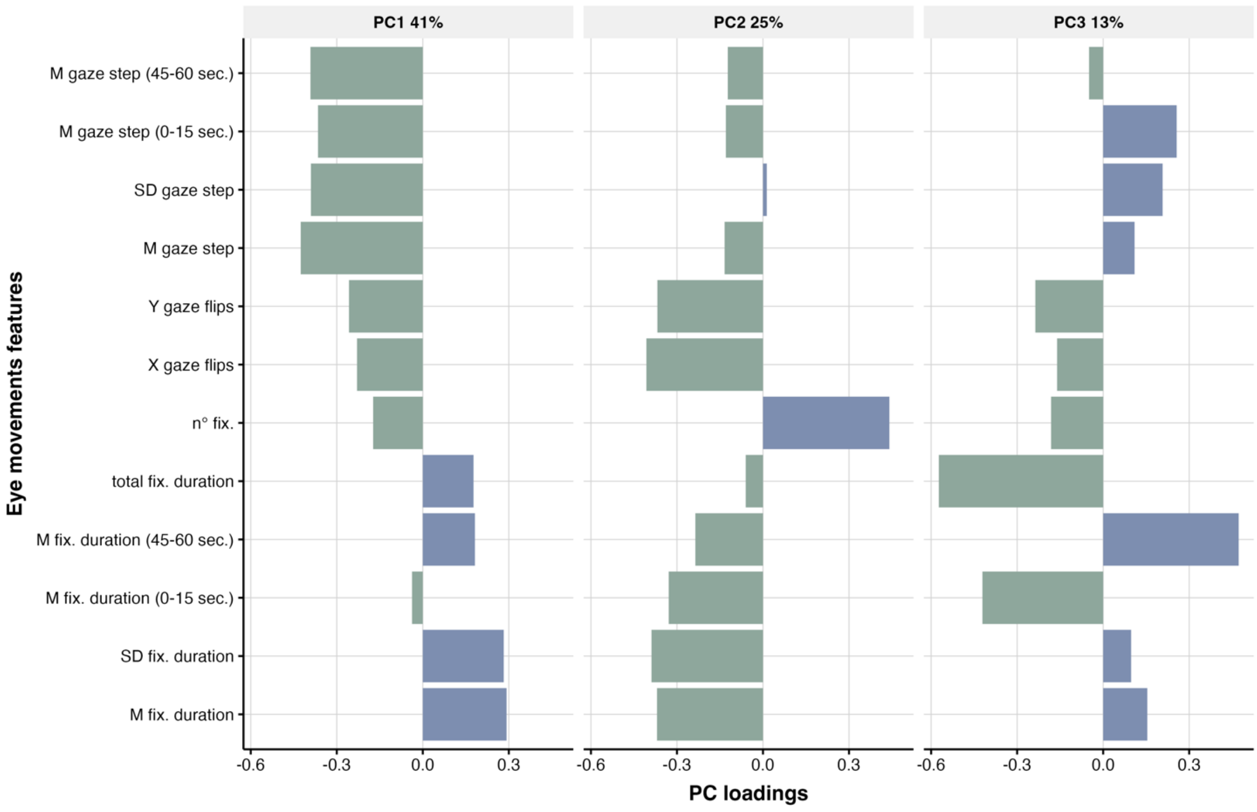

Fig. S1. Principal components loadings of the extracted eye movements statistics during pre-FV. The three horizontally stacked bar plots represent the first three principal components, ordered per variance explained. Fix. = Fixation; M = Mean; SD = standard deviation.

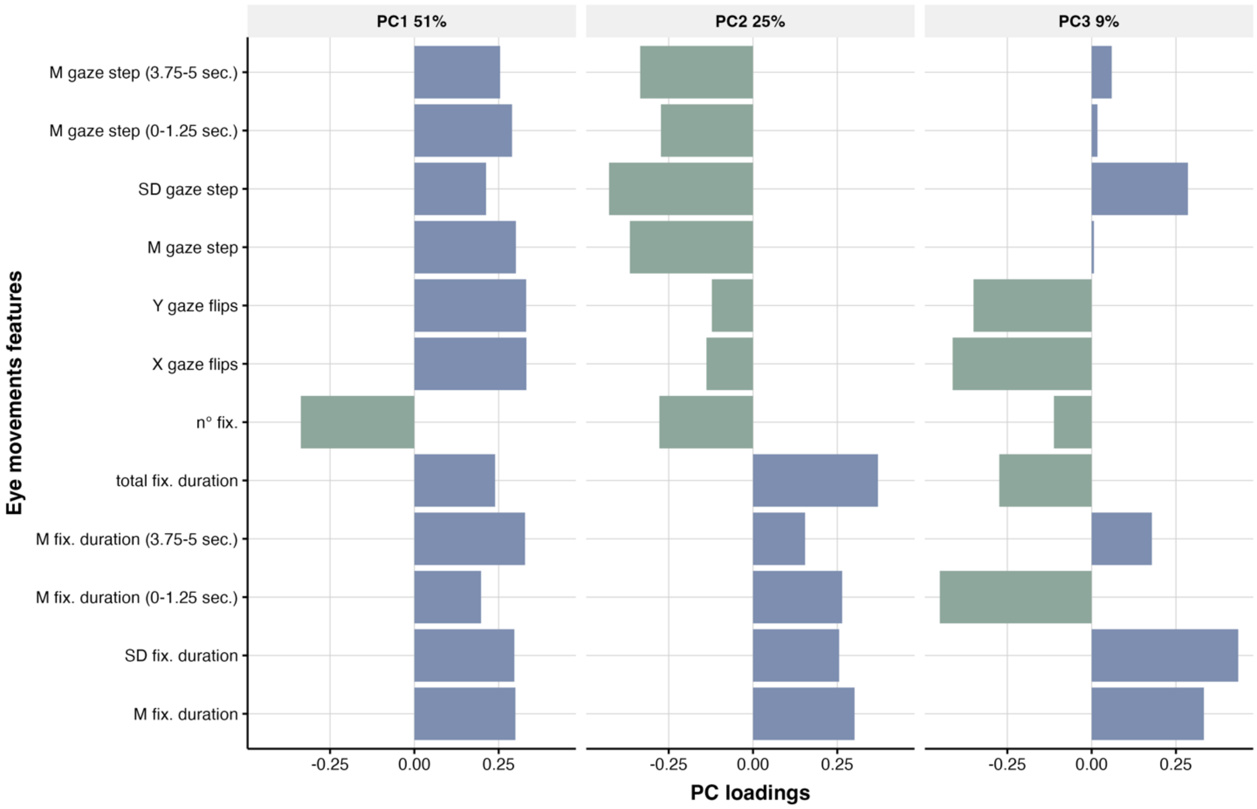

Fig. S2. Principal components loadings of the extracted eye movements statistics during pre-IVT. The three horizontally stacked bar plots represent the first three principal components, ordered per variance explained.

Table S1.

Preregistration deviations

#	Details		Original wording	Deviation description	To what extent is this a deviation from the preregistered plan?	Judgment of impact
1	Type	Analyses	"One sample t-test with the mean horizontal gaze difference (one-sided) for IVT."	Upon data inspection of the 30 fixation's shifts of each participant we found that 9 out of 40 participants showed non normal distributions according to the D'Agostino-Pearson Omnibus test (alpha = 0.05), which assesses deviations in skewness and kurtosis. Thus, we opted for the median instead of the mean to summarize the individual distributions, as it gives less biased centrality measures when the distribution is asymmetric.	Minor	As this deviation was necessary given the inappropriateness of the statistic for the data at hand, the risk of bias should be minimal. To further validate the results obtained we performed a robustness check. Concretely, we performed a Wilcoxon signed rank test on each individual distribution and then computed the respective Z scores and r effects sizes. The average effect size across the whole sample was small (mean r = 0.22), in line with the small Cohen's d effect size found in the main analysis. The individual distributions and the robustness check are documented in the publicly available analyses scripts (https://osf.io/ws5dx/).
	Reason	Plan not possible				
	Timing	After data access				

SI References

Hooge, I. T. C., Niehorster, D. C., Nyström, M., Andersson, R., & Hessels, R. S. (2022). Fixation classification: How to merge and select fixation candidates. *Behavior Research Methods*. https://doi.org/10.3758/s13428-021-01723-1
